# Advances in the Management of Central Nervous System Metastases in Non-Small Cell Lung Cancer

**DOI:** 10.3390/cancers15030844

**Published:** 2023-01-30

**Authors:** Angelica D’Aiello, Emily Miao, Haiying Cheng

**Affiliations:** 1 Department of Oncology, Montefiore Medical Center, Albert Einstein College of Medicine, Bronx, NY 10461, USA; 2 Albert Einstein College of Medicine, Bronx, NY 10461, USA

**Keywords:** brain metastases, central nervous system (CNS) metastases, immunotherapy, non-small cell lung cancer (NSCLC), precision oncology, targeted therapy

## Abstract

**Simple Summary:**

Central nervous system (CNS) metastases are common and challenging to manage among patients with non-small cell lung cancer (NSCLC). Here we provide an overview on lung cancer CNS metastases, including our evolving understanding of the genetic landscape, treatment updates, and future directions.

**Abstract:**

Central nervous system (CNS) metastases are common among patients with non-small cell lung cancer (NSCLC). While the presence of brain metastases has historically portended poor prognosis, recent advances in local and systemic therapies have greatly improved outcomes for NSCLC patients with CNS involvement. Stereotactic radiology surgery (SRS) has emerged as an effective radiotherapy technique with fewer toxicities compared to whole brain radiotherapy (WBRT). Furthermore, multi-generation tyrosine kinase inhibitors (TKIs) with CNS overall response rates (ORR) of up to 70–80% are now an accepted first-line approach for a subset of advanced NSCLC patients with targetable molecular alterations. In addition, while the CNS was once considered an immunologic sanctuary site, growing evidence shows that immune checkpoint inhibitors (ICIs) can induce durable responses in brain metastases as well. Ongoing efforts to optimize CNS metastases management are necessary to refine multimodal treatment approaches and develop new therapeutics with better CNS penetrance.

## 1. Introduction

**Figure 1:**
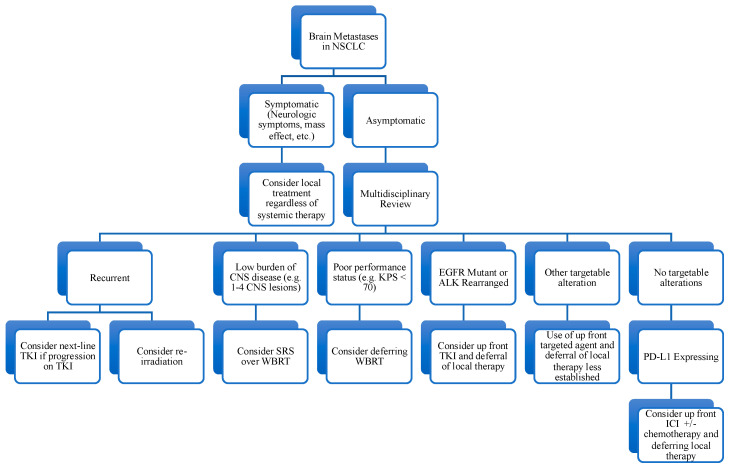
Decision tree for management of brain metastases in non-small cell lung cancer. Abbreviations: non-small cell lung cancer (NSCLC); central nervous system (CNS); Karnofsky Performance Status (KPS); tyrosine kinase inhibitor (TKI); stereotactic radiosurgery (SRS); whole brain radiotherapy (WBRT); immune checkpoint inhibitor (ICI).

Among patients with malignancy, lung cancer represents the most common primary tumor associated with the central nervous system (CNS) metastases, accounting for up to 50% of the cases [1]. Roughly 10% of patients newly diagnosed with lung cancer have existing CNS involvement, and 20–40% will go on to develop brain metastases [2]. The incidence of CNS metastases is higher still among patients with non-small cell lung cancer (NSCLC) and driver oncogenes, especially EGFR mutations and ALK rearrangements, in whom up to 60% will develop brain metastases [3,4,5]. Given such a high prevalence of CNS involvement among NSCLC patients, it is of paramount importance to optimize treatment strategies for brain metastases. Both local therapies such as radiotherapy and surgery and systemic treatments with CNS activity including targeted therapy and immunotherapy are currently established components of management. The objective of this article is to review recent advances in the management of brain metastases including novel radiotherapy techniques, targeted agents with demonstrated CNS activity, evidence for immune checkpoint inhibitors, and strategies for treating leptomeningeal disease. Given the complex nature of managing CNS metastases in NSCLC, often requiring multidisciplinary input, we also propose here a simplified decision tree that may be of use to a treating clinician (Figure 1).

## 2. Local Treatments

### 2.1. Radiotherapy

In the past, whole brain radiotherapy (WBRT) was the standard radiation approach for the treatment of brain metastases. Due to toxicities associated with WBRT, especially cognitive ones, treatment has increasingly shifted to utilizing stereotactic radiosurgery (SRS) instead. Both radiation options have several important differences in administration, side effects, and efficacy.

WBRT typically involved the administration of 20–37.5 Gy delivered over 5–15 fractions. Although WBRT improved regional CNS control, it did not prolong overall survival (OS) and was associated with neurocognitive decline compared to SRS [6,7,8]. In addition, the QUARTZ study found that in patients with NSCLC and CNS involvement with Karnofsky performance status less than 70 who were not candidates for SBRT or surgery, observation alone did not show any differences in OS or quality of life (QOL) compared to WBRT suggesting that supportive care alone may be appropriate in patients with poor baseline prognoses [9]. One study comparing SRS alone versus SRS plus WBRT showed that most patients who received WBRT develop measurable cognitive deterioration as soon as three months following completion of the therapy, a rate significantly higher than those who received SRS alone (91.7% versus 63.5%, respectively, with a 28.2% difference (90% CI, 14.4 to 41.9%, *p* < 0.001)) [6]. To address the cognitive toxicities of WBRT, the NRG CG001 trial studied the application of hippocampal-avoidance (HA) WBRT plus the N-methyl-D-aspartate receptor antagonist memantine, which was associated with lower rates of cognitive failure compared to standard WBRT plus memantine (adjusted HR, 0.74, 95% CI, 0.58 to 0.95, *p* = 0.02) [10].

SRS is typically administered in 1–5 fractions and has been shown to achieve up to 80–90% local control rates comparable to WBRT for up to 15 brain metastases [11]. Most trials comparing SRS to WBRT, however, included patients with no more than four brain metastases [12]. Compared to SRS plus WBRT, SRS alone has similar outcomes in terms of OS with improved QOL and fewer cognitive adverse effects [13].

Although SRS has an improved tolerability profile and excellent local control rates, radiation necrosis is a late toxicity of concern, especially for tumors with large size, typically defined as greater than 2 cm [14]. Radiation necrosis following treatment can present a diagnostic challenge, as it is often difficult to distinguish between such radiotherapy-related treatment changes and disease recurrence. Once radiation necrosis is suspected, the standard of care for symptomatic patients includes steroids, though the optimal treatment regimen has not been established. For severe symptomatic cases of radiation necrosis, a small placebo-controlled randomized controlled trial of bevacizumab has demonstrated improvements in neurologic symptoms and follow-up imaging assessments [15].

### 2.2. Surgery

In the setting of oligometastatic CNS metastases, surgery represents a potentially curative approach [16]. Other indications for surgery include large brain metastases with mass effect, significant edema, or those that are symptomatic. Given the potential for recurrence, adjuvant radiotherapy following resection is the standard of care [17].

## 3. Systemic Therapy

### 3.1. Molecular Targeted Therapy

In the era of precision oncology, an individualized approach to patient care based on specific molecular alterations is increasingly utilized, nowhere more so than in NSCLC. Currently, there are nine main biomarkers with FDA-approved targeted therapies with varying degrees of CNS activity, including alterations involving multiple EGFR mutations, ALK rearrangement, ROS1 rearrangement, KRAS G12C, BRAF V600E, NTRK1/2/3 fusions, RET rearrangement, and ERBB2 (HER2). Despite demonstrated CNS activity of many of these agents in studies leading to approval, limitations exist—most of these trials evaluated CNS outcomes only as secondary endpoints or used post hoc analyses, thus constraining our understanding of the efficacy of these treatments for brain metastases. In addition, among such trials, there is significant heterogeneity in terms of the extent of CNS involvement, pretreatment with radiotherapy, and specific CNS endpoints evaluated, further limiting inter-trial comparisons.

The frequency of targetable alterations varies somewhat depending on early versus advanced-stage disease, with EGFR followed by KRAS being the most frequent driver mutations in the metastatic setting [18]. Among patients who develop CNS involvement, specifically, EGFR mutations and ALK rearrangements are particularly common, possibly related to extended survival seen in such patients following the arrival of TKIs, therefore allowing more time to develop brain metastases [3,4]. Detection of driver mutations among patients with CNS metastases may also differ depending on the origin of the specimen tested (lung, brain, blood, or cerebral spinal fluid). For example, a recent retrospective analysis investigating the genomic profiles of NSCLC patients with brain metastases showed that in a series of paired lung and brain biopsies samples from individual patients with time between collections dates ranging from 2 days to 5 years (median = 440 days), 85% had at least one additional genomic alteration detected from the brain compared to the lung sample [19]. Various hypotheses for such genetic heterogeneity have been postulated including the proliferation and acquired metastatic potential of preexisting subclonal populations within primary tumor sites over time as well as increasing genetic complexity arising secondary to exposure to targeted therapies [20]. While liquid biopsy has shown concordance with tissue biopsy, leading to its widespread use in the advanced setting, the blood-brain barrier (BBB) may limit the utility of circulating tumor DNA (ctDNA) for assessing the genomic landscape of NSCLC metastasized to the CNS [21]. In addition, several studies have shown that analyzing ctDNA from the CSF may be a more sensitive and specific source for detecting genomic alterations compared to blood-derived ctDNA [22,23,24]. Based on such findings, testing CSF for ctDNA may be a beneficial adjunct to standard blood-based ctDNA assays that could aid in identifying targetable molecular alterations as well as identifying resistance mechanisms in patients on targeted therapy.

As detailed in Table 1, targeted therapies currently approved for oncogene-driven NSCLC have shown varying degrees of CNS activity. The most robust evidence for CNS activity comes from multiple phase III trials investigating the third-generation EGFR inhibitor osimertinib and newer-generation ALK TKIs. For other targeted treatments, evidence is less compelling as many trials involving these agents were not designed to collect or report CNS outcomes.

The third-generation EGFR inhibitor osimertinib showed improved CNS activity when compared to platinum-based chemotherapy (ORR 40% versus 17%) in the Phase III AURA 3 trial for EGFR-mutated patients who had progressed on a prior TKI [25]. In addition, the Phase III FLAURA trial demonstrated osimertinib’s superiority to the standard of care TKI with CNS ORR 66% versus 43% in EGFR-mutated treatment naïve NSCLC patients [26]. For those patients harboring other less common EGFR alterations (S7681, L861Q, and G719X) data regarding CNS activity of TKIs is lacking as patients with CNS metastases were excluded from the largest study of uncommon EGFR alterations consisting of a post hoc pooled analysis of three trials investigating afatinib versus platinum-based chemotherapy [52]. Among treatment-naïve patients with EGFR Exon 20 insertion and brain metastases, mobocertinib showed a CNS ORR of only 25%, suggesting limited intracranial activity [53]. While amivantamab, an EGFR-MET bispecific antibody, was approved for the treatment of EGFR Exon 20 insertion NSCLC based on the Phase I CHRYSALIS trial, patients with active or untreated brain metastases were excluded from the study, and CNS monitoring was not required limiting interpretation of CNS activity [54].

EGFR inhibition has also been evaluated in combination with VEGF inhibition. The Phase III randomized NEJ026 trial evaluated first-line erlotinib plus the VEGF inhibitor bevacizumab versus erlotinib alone in patients with EGFR-mutated advanced nonsquamous NSCLC [27]. Among patients with brain metastases, there was no difference in OS between treatment groups, though authors advise cautious interpretation of these results, as this subgroup analysis was underpowered. No other CNS-specific outcomes were included. The recombinant monoclonal antibody targeting VEGF receptors, ramucirumab, was also studied in combination with erlotinib in the RELAY trial; however, patients with CNS metastases were excluded [55].

In the setting of ALK-rearranged NSCLC, multiple trials have demonstrated excellent CNS activity of second and third-generation ALK inhibitors compared to first-generation crizotinib. For patients who progressed on crizotinib with baseline CNS involvement, the second-generation alectinib showed CNS ORR of 57% with 43% of patients achieving a complete response (CR) [28]. In addition, alectinib showed superior CNS outcomes compared to crizotinib in the upfront setting, with CNS ORR up to 74.4% versus crizotinib 24.3% in patients with untreated asymptomatic brain metastases [29], (and delayed time to CNS progression in those without brain metastases (HR 0.19, *p* = 0004) compared to crizotinib [30]. Similar findings were seen with additional second-generation ALK inhibitors, brigatinib and ceritinib. Finally, the third-generation ALK inhibitor lorlatinib has also shown CNS activity both in treatment naïve settings with CNS ORR up to 82% and in patients previously treated with non-crizotinib ALK inhibitors and/or chemotherapy [35,36].

For patients with ROS1 rearranged NSCLC, entrectinib yields a CNS ORR of up to 79% in patients with measurable brain metastases [38]. Lorlatinib, which is both an ALK and ROS1 inhibitor, yields a somewhat lower CNS activity compared to its performance in the ALK setting with a CNS ORR of 64% in treatment naïve and 50% in patients previously treated [39]. Another ROS inhibitor currently under investigation, repotretinib, may also have CNS activity with a CNS ORR of 100% in treatment naïve patients with brain metastases though small patient size (n of 3) limits interpretation [40].

Recently, KRAS G12C has emerged as a targetable alteration with the KRAS inhibitors sotorasib and adagrasib. CNS outcomes were not assessed in the Phase II CodeBreak 100 trial evaluating sotorasib as patients with brain metastases were excluded [41]; however, the Phase I KRYSTAL-1 trial investigating adagrasib allowed patients with treated CNS metastases and showed an intracranial ORR of 33% [39]. A follow-up report by Sabari and colleagues found that adagrasib achieved steady concentrations in the CSF of two patients with untreated brain metastases enrolled in the KRYSTAL-1 trial, with one patient achieving a partial response and the other with stable disease after two cycles of treatment. In addition, the authors showed that adagrasib penetrated the CSF and extended survival in preclinical mouse models of KRAS G12C-mutant NSCLC. Together, these findings further support that adagrasib has clinical activity against brain metastases in KRAS G12C-mutant NSCLC [56].

RET inhibition with selpercatinib also demonstrated impressive CNS activity in the LIBRETT0-001 trial, with a CNS ORR of 81.8% among 22 patients with measurable brain mets [50]. In addition, the RET inhibitor pralsetinib showed CNS activity but to a lesser extent than selpercatinib with a CNS ORR of 56% and only evaluable in 9 patients [49]. Additional therapies targeting BRAF V600E, NTRK gene fusions, and MET Exon Skipping have also demonstrated CNS activity, though small data sets limit conclusions from existing trials [43,44,45,46,47,48]. Finally, the recently approved ERBB2 (HER2) therapy trastuzumabderuxtecan, while investigated in patients with brain metastases, did not report upon specific CNS outcomes and additional study is necessary to assess its CNS activity [51].

### 3.2. Systemic and Local Therapy Combinations

There is limited and mixed data comparing upfront TKIs alone versus the addition of radiotherapy for the treatment of CNS metastases. While a prior landmark study suggested that upfront EGFR-TKI and deferral of radiotherapy for brain metastases were associated with inferior survival, the study focused on the first-generation EGFR inhibitor erlotinib which has limited CNS activity compared to the third-generation osimertinib [57]. The BRAIN trial compared icotinib upfront with WBRT on progression to the combination of WBRT and chemotherapy in patients with EGFR-mutant NSCLC and found that patients receiving icotinib upfront with WBRT on progression had better intracranial PFS but no difference in OS [58]. A more recent multi-institutional study found no significant differences between upfront RT + TKI vs. TKI alone for patients with EGFR- and ALK-positive NSCLC, suggesting that RT can be deferred during the progression of the disease [59]. Recent CNS metastases consensus guidelines published by ASCO/ASTRO recommend consideration of upfront TKI for NSCLC with brain metastases in the setting of EGFR and ALK alterations but do not comment on the use of TKIs for other targetable alterations [12]. Despite the appeal of a monotherapy TKI approach, one must consider the varying potential for long-term control with SRS weighed against some TKI with median durations of CNS response lasting in the range of 9–12 months only (Table 1). In addition, while there is limited data on toxicities from combined approaches of SRS and TKIs, there is the potential for an increased risk of radionecrosis. There is a continued need for prospective trials to compare the efficacy and adverse effects of combination approaches versus TKI alone, and while we await such results, recent guideline statements call for a personalized approach to therapy including a review in multidisciplinary tumor boards to determine individual treatment plans [12].

### 3.3. CNS Progression on TKIs

Yet another challenge in the era of targeted therapy is the approach to CNS progression while on TKI. In the absence of a repeat brain biopsy, it may be difficult to establish whether such CNS progression represents resistance versus suboptimal CNS penetrance for a given targeted agent. While ctDNA may be utilized to detect resistance mechanisms, such as the development of a new alteration, it is neither sensitive nor specific in the setting of brain metastases. Given the diagnostic challenges of establishing TKI resistance as the mechanism for CNS progression, there are several potential options for management. One option would be to continue the TKI with the addition of local therapy given data showing an extended duration of response with the addition of TKIs [60]. Dose escalation is yet another accepted strategy backed by evidence [61]. Finally, one may also consider switching TKIs. For example, in the setting of ALK and ROS inhibitors, later generations are known to have superior CNS response rates (Table 1).

### 3.4. Immunotherapy

Over the last decade, immune checkpoint inhibitors (ICIs) have revolutionized the treatment landscape for patients with advanced NSCLC, particularly in those without molecularly targetable mutations [62]. Blockade of cytotoxic T-lymphocyte-associated protein 4 (CTLA-4), programmed death protein-1 (PD-1), and its ligand has led to improved survival outcomes for these patients. Historically, the CNS was considered an immunologic sanctuary site, but evidence showing that ICIs can induce durable responses in brain metastases challenges this long-held notion [63,64,65,66]. Compared with gliomas, for which outcomes from ICIs have been disappointing, brain metastases in solid tumors including NSCLC appear to be to have higher infiltrations of tumor-infiltrating lymphocytes (TILs), which are important for immunotherapy efficacy [67,68,69]. In matched comparisons of primary lung and CNS metastases in NSCLC, higher PD-L1 expression but lower TILs have been observed in brain metastases, perhaps contributing to variable CNS activity of immunotherapy [69,70]. Further studies are necessary to reveal to what extent the effectiveness of immunotherapy for brain metastases relies upon intracranial immune function versus migrating immune cells from the periphery.

Current data on the efficacy of ICIs in lung cancer brain metastases remain limited, as most clinical trials have historically excluded patients with active or untreated brain metastases [71]. To date, the strongest evidence of ICI efficacy for the treatment of active CNS metastases comes from a Phase II trial of pembrolizumab by Goldberg et al. [72]. The study included patients with non-oncogene-driven, advanced NSCLC with untreated, asymptomatic brain metastases (5–20 mm) not requiring steroids. Overall, 42 patients with brain metastases were included. Among patients with PD-L1 ≥ 1%, the CNS ORR was 29.7% (11/37), with a median CNS DOR of 5.7 months. PFS and OS were 1.9 months and 9.9 months, respectively. There were no responses in the PD-L1 negative or unknown cohort. Several pooled analyses and retrospective studies have similarly confirmed these CNS outcomes with pembrolizumab, showing intracranial ORR ranging roughly 15–35% regardless of PD-L1 expression but exceeding 50% in patients with PD-L1 ≥ 50% [73,74,75,76]. Other key studies of ICI monotherapy can be found in Table 2.

Dual ICIs have also been evaluated in patients with NSCLC and CNS metastases. A post hoc analysis of Checkmate-227 evaluating nivolumab plus ipilimumab versus chemotherapy showed that combination ICI appeared to provide similar survival benefits in patients with and without treated baseline CNS metastases [81]. While prospective data ultimately remains limited, there are several ongoing clinical trials evaluating the efficacy of ICIs for untreated brain metastases (NCT02681549, NCT 02886585, NCT03526900).

Given that ICI monotherapy provides a relatively modest response in NSCLC brain metastases, combination strategies including ICIs with chemotherapy or radiation have been of interest. There is growing evidence that radiation synergizes with immunotherapy by increasing the visibility of tumor antigens and modulating the tumor microenvironment [82,83]. Furthermore, a phenomenon known as the abscopal effect—the ability for local radiation to induce an antitumor response at sites not subjected to radiation—has been observed with the combination of ICI and radiotherapy [84]. While prospective studies in this domain are lacking, retrospective studies have reported excellent local control and superior OS outcomes when ICIs are combined with radiation. In a meta-analysis of 19 studies, the combination of ICI with CNS radiation demonstrated superior OS (HR 0.77, 95% CI: 0.71–0.83) without any increase in neurologic toxicity compared to CNS radiation alone for NSCLC [85]. Similarly, another meta-analysis (n = 534 patients, 1570 brain metastases) reported improved 1-year OS (64.6% vs. 51.6%) and 1-year local control (89.2% vs. 67.8%) in patients who received ICI with SRS compared to non-concurrent therapy [86].

Like radiation therapy, chemotherapy may also synergize with ICIs [87]. Recent data suggest that combining ICIs with chemotherapy improves survival regardless of PD-L1 expression or the presence of brain metastases at baseline [88]. In KEYNOTE-189, first-line pembrolizumab with pemetrexed-platinum chemotherapy significantly improved median OS (22.0 vs. 10.7 months and HR 0.56, 95% CI: 0.45–0.70), and PFS (9.0 vs. 4.9 months and HR 0.45, 95% CI: 0.40–0.58), compared to chemotherapy alone [88]. A pooled analysis of several key trials (n = 1298 patients, 171 brain metastases) also supports these findings [89]. In patients with brain metastases who received pembrolizumab with chemotherapy, median OS and PFS were 18.8 months and 6.9 months, respectively. Median systemic DOR was also longer (11.3 vs. 6.8 months) compared to the chemotherapy only group.

Yet another emerging area of interest is the combination of ICI with antiangiogenic agents. It is hypothesized that antio-angiogenic agents may facilitate the efficacy of immunotherapy by normalizing aberrant tumor vasculature, decreasing tumor-promoting hypoxia, and increasing the accessibility of immune cells to access the tumor microenvironment [90]. The IMpower150 trial randomized patients 1:1:1 to receive atezolizumab + bevacizumab + carboplatin/paclitaxel (ABCP), atezolizumab + carboplatin/paclitaxel (ACP), or bevacizumab + carboplatin/paclitaxel (BCP) (NCT02366143). Further exploratory analyses of IMpower150 among patients with baseline brain metastases found that the ABCP combination delayed the time to development of new brain metastases (HR 0.68 for ABCP versus BCP, and HR 1.55 for ACP versus BCP) [91]. In addition, a phase 1b trial of the PD-1 inhibitor sintilimab combined with the multi-target TKI with antiangiogenic action showed promising CNS activity in the frontline setting with all four patients with baseline asymptomatic brain metastases achieving IRR CR [92]. Further efforts are necessary to investigate the role of combination ICI with antio-angiogenic therapies without or without chemotherapy.

## 4. Leptomeningeal Disease

Leptomeningeal metastasis (LM) occurs when tumor cells disseminate and spread to the meninges, which include the pia and arachnoid mater. Malignant cells may reach the leptomeningeal space, a so-called immune-privileged site through direct extension from brain metastases or via the hematogenous or lymphatic spread. Throughout the course of disease among patients with advanced NSCLC, LM occurs in 3–5% of patients and up to 10% in patients with EGFR-mutated and ALK-rearranged NSCLC [93,94]. The mechanisms underlying the higher incidence of LM disease among patients with EGFR and ALK alterations may be multifactorial. One hypothesis is that longer survival in patients on TKIs may provide more time for metastatic malignant cells to overcome the blood-brain barrier. An alternative explanation is insufficient penetration of earlier generation TKIs into the CSF, allowing LM to develop. Nonetheless, LM is a devastating end-stage complication of NSCLC as OS remains in the order of several weeks to months [93,95].

Diagnosis of LM remains challenging and includes a combination of clinical manifestations, cerebrospinal fluid (CSF) cytology, and neuroimaging findings [96]. The current gold standard is a positive CSF cytology via lumbar puncture; however, the sensitivity is only 50% at initial sampling and increases to 75–85% on subsequent CSF analyses [97]. Similarly, brain and spine magnetic resonance imaging (MRI) has its shortcomings, as 20–30% of patients with LM have normal or false-negative MRIs [95]. New emerging approaches such as CSF liquid biopsy allow for the analysis of tumor components in CSF including circulating tumor cells (CTCs) and ctDNA [94,98]. Studies have shown that quantification of CTCs in CSF has been shown to be more sensitive than conventional cytology combined with neuroimaging [99,100]. Additionally, circulating tumor DNA in CSF may also be used as an adjunct to detect LM, specifically in cases that cannot be detected by conventional methods [22].

LM is more common in patients harboring EGFR mutations or ALK rearrangements [93,94]. However, there is no standard regimen regarding management. In patients who have progressed on first- or second-generation TKI with LM confirmed via cytology, the BLOOM (Phase I) study showed osimertinib 160 mg to have an LM ORR of 62% (95% CI: 45–78) and a median duration of response of 15.2 months (95% CI: 7.5 to 17.5) [101]. In the study, LM ORR was assessed by neuroradiologic blinded central independent review (BICR) according to Response Assessment in Neuro-Oncology LM radiologic criteria as well as other reviews according to the RANO-LM working group criteria which incorporates a standard neurologic exam and CSF cytology or flow cytometry [102]. In another study of patients who developed LM on osimertinib, dose escalation from 80 mg to 160 mg has also demonstrated modest local control, with a median of 5.8 months (95% CI: 1.7–9.1) [103]. ALK rearrangements are less common as compared to EGFR mutations, and consensus regarding the management of LM in this population is limited by the paucity of existing data. A few case reports have described alectinib and brigatinib as successful salvage options for patients who developed LM on crizotinib or ceritinib [104,105]. Moreover, alectinib dose escalation from 600 mg to 900 mg twice daily has been reported to overcome incomplete ALK inhibition in the CNS and prolong the durability of responses in two patients with LM [106]. Perhaps dose escalation approaches can be employed for the treatment of LM in NSCLC harboring other driver oncogenes, but larger prospective studies are needed.

Other treatments for LM include radiotherapy, systemic and intrathecal chemotherapy, and adjunctive bevacizumab. Radiotherapy is especially recommended for symptomatic sites associated with LM [107]. For example, lumbosacral spine radiotherapy may be utilized for patients with cauda equina syndrome, while WBRT may be beneficial for patients with isolated cranial neuropathies [108]. Recently, proton craniospinal irradiation has emerged as a potentially beneficial treatment strategy for patients with LM and adequate functional status [109,110]. Systemic pemetrexed has also been studied for the treatment of LM, and in one retrospective analysis, pemetrexed use after LM was independently associated with a longer median post-LM survival (13.7 months, 95% CI: 4.1–23.2) compared to those without pemetrexed use after LM (median 4.0 months, 95% CI: 2.2–5.7) [111]. Intrathecal chemotherapies have also demonstrated modest efficacy with a median OS of 6 months [112]. Bevacizumab, an anti-VEGF inhibitor, has also been used for the treatment of LM as an adjunct alongside TKIs including osimertinib or lorlatinib. When bevacizumab is added to osimertinib and lorlatinib, median LM PFS was reported to be 9.3 months (95% CI: 8.2–10.4) and 5–9 months, respectively [113,114]. Finally, there is limited data regarding the safety and efficacy of ICIs in patients with LM. Two phase II trials evaluated pembrolizumab for the treatment of LM in patients with solid tumor malignancies with median OS ranging from 2.9 to 3.6 months [115,116]. Altogether, only 3 of 33 patients had NSCLC; thus, it is difficult to draw any definitive conclusions.

## 5. Directions for Future Research

Continued progress in the treatment of NSCLC metastatic to the brain will depend upon a better understanding of the biological basis of CNS metastases, improved trial design with deliberate inclusion of CNS-specific outcomes, and the development of novel therapeutics. While the basis for lung cancer metastasis to the CNS is yet to be fully elucidated, various preclinical research efforts have investigated how novel agents might be applied to minimize metastasis. One such study showed that the compound cephalomannine exerts inhibitory effects in hypoxic lung cancer cells, reducing cell migration, and possibly metastatic potential [117]. Other ongoing areas of investigation aim to identify molecular alterations that may be specifically associated with CNS metastases, and potentially targetable. For example, the genomic profiles of NSCLC patients have been shown to differ in those with brain metastases compared to those without, with enrichment in alterations such as KRAS, NFKBIA, and RICTOR among patients with CNS involvement [19,118]. Furthermore, a preliminary report using an in vivo mouse model of NSCLC showed that RICTOR knockdown inhibits lung cancer tumor growth and spread in the brain, suggesting that RICTOR may represent an important therapeutic target for treating or preventing brain metastases [107]. Further efforts are necessary to identify other targets and treatments for NSCLC CNS metastases.

## 6. Conclusions

With the advent of improved radiotherapy approaches such as SRS and the multiple generations of TKIs with increasing CNS penetrance, the treatment options, and prognoses for patients with CNS metastases have vastly improved. Still, many unanswered questions remain. Current consensus guidelines emphasize tailoring treatment plans based on individual patient characteristics and multidisciplinary review. In the absence of a “one-size-fits-all” approach, however, clinicians ultimately lack clear guidance for the first-line treatment of brain metastases beyond locoregional options. In addition, many clinical trials evaluating novel targeted agents and immune checkpoint inhibitors have either excluded patients with symptomatic brain metastases or failed to include CNS-specific outcomes, ultimately limiting the interpretation of efficacy for patients with brain metastases. Continued progress is required to standardize treatment approaches, design clinical trials with the ability to detect CNS-specific benefits, and develop more effective therapeutics.

## Figures and Tables

**Table 1:** Efficacy of Targeted Therapies in Non-small Cell Lung Cancer (NSCLC) with Central Nervous System (CNS) Metastases.

Trial	Phase	Systemic Therapy	Setting/CNS Inclusion Criteria	Sample Size (with CNS Disease)	Key Results (CNS Outcomes)	Publication Year	Reference
*EGFR* Typical Mutations (Exon 19 Deletions and Exon 21 L858R Mutations)							
AURA 3	III	Osimertinib (Osi) vs. Platinum-based chemotherapy (chemo)	T790M+ NSCLC patients with asymptomatic, stable BM following POD with prior EGFR-TKI	Measurable BM: Osi (n = 30) vs. Chemo (n = 26) All BM: Osi (n = 75) vs. Chemo (n = 41)	All BM: CNS ORR, Osi (40%) vs. chemo (17%), OR 3.24 (*p* = 0.014) CNS median DOR: Osi (8.9 mo) vs. Chemo (5.7 mo) Median iPFS: Osi (11.7 mo) vs. Chemo (5.6 mo) Median PFS: Osi (8.5 mo) vs. Chemo (4.2 mo); HR 0.32; 95% CI: 0.21–0.49)	2018	[25]
FLAURA	III	Osimertinib (Osi) vs. SOC EGFR-TKI	Treatment-naïve EGFR-mutated NSCLC Patients with asymptomatic or stable BM Symptomatic pts must have stable neurologic status ≥ 2 weeks following definitive local therapy	Measurable BM: Osi (n = 22), SOC EGFR-TKI (n = 19) All BM: Osi (n = 61) vs. SOC EGFR-TKI (n = 67)	All BM CNS ORR: Osi (66%) vs. SOC EGFR-TKI (43%) CNS Median DOR: Osi (15.2 mo) vs. SOC EGR-TKI (18.7 mo) Median iPFS: Osi (NR) vs. SOC EGFR-TKI (13.9 mo) PFS at 18 months: 58% (95% CI: 40–72) in osimertinib group OS (BM subgroup): HR 0.83 (0.53–1.30)	2018	[26]
***EGFR* Exon 20 Insertion**							
NCT02716116	I, II	Mobocertinib	Previously treated Patients with active and symptomatic BM included	N = 12	CNS ORR: N/A Overall CNS ORR: 25%, among the patients with BM	2021	[25]
**Combined *EGFR* and *VEGF* Inhibition**							
NEJ026	III	Erlotnib vs. Erlotinib/bevacizumab	EGFR-positive advanced NSCLC Patients with BM requiring anti-edema drugs excluded	All BM: Erlotnib (n = 36) vs. Erlotinib/bevacizumab (n = 36)	OS (BM subgroup): HR 0.839 (0.432–1.629)	2021	[27]
***ALK* Rearrangement**							
NCT02075840	II	Alectinib	Disease progression on crizotinib Patients with stable, treated brain and/or leptomeningeal metastases or asymptomatic untreated brain and/or leptomeningeal metastases were allowed	All BM (n = 84)	CNS ORR 57% (95% CI, 39% to 74%) CNS disease control rate 83% (95% CI, 74% to 91%), CNS DOR was 10.3 months (95% CI, 7.6 to 11.2 months) CNS CR in 43% of patients with baseline CNS metastases	2016	[28]
ALEX	III	Alectinib vs. Crizotinib	Treatment of naïve patients with asymptomatic BM (treatment with local therapy allowed)	All BM: Alectinib (n = 64) vs. Crizotinib (n = 58) Measurable BM: Alectinib (n = 21) vs. Crizotinib (n = 22)	All BM: CNS ORR, Alectinib (36%) vs. Crizotinib (28.6%) with prior radiation; Alectinib (74.4%) vs. Crizotinib (24.3%) without prior radiation CNS Median DOR: Alectinib (NR) vs. Crizotinib (17.3 mo)	2018	[29]
J-ALEX	III	Alectinib vs. Crizotinib	Treatment naïve, or failed one line of chemotherapy regimen Asymptomatic BM (treated or untreated allowed)	Alectinib (n = 14) vs. Crizotinib (n = 29)	Time to CNS progression (alectinib superior): HR = 0.51, *p* = 0.2502 with baseline BM vs. HR = 0.19, *p* = 0.0004 without baseline BM 1-year CNS Cumulative Incidence Rate: Alectinib (5.9%) vs. Crizotinib (16.8%)	2018	[30]
ALTA	II	Brigatinib (Arm A—90 mg QD, Arm B—180 mg QD)	Disease progression on crizotinib	All BM: 154	CNS ORR 42% in arm A and 67% in arm B	2018	[31]
ALTA-1L	III	Brigatinib vs. Crizotinib	Treatment-naïve Patients with asymptomatic or stable BM not requiring steroids or anticonvulsive therapy 7 days prior to randomization	All BM: Brigatinib (n = 47) vs. Crizotinib (n = 49) Measurable BM: Brigatinib (n = 18) vs. Crizotinib (n = 23)	Measurable BM: CNS ORR, Brigatinib (78%) vs. Crizotinib (26%), OR 11.67 (*p* = 0.0014) Median iPFS: Brigatinib (24 mo) vs. Crizotinib (5.6 mo) in patients with baseline BM; 32.3 mo (Brigatinib) vs. Crizotinib (NR) in patients without baseline BM	2020	[31,32]
ASCEND-4	III	Ceritinib vs. Platinum-based chemotherapy	Treatment-naïve Patients with asymptomatic brain metastases (prior treatment allowed)	All B: Ceritinib (n = 59) vs. Chemotherapy (n = 80)	CNS ORR ceritinib (72.7%) vs. chemotherapy (27.3%) Median CNS DOR ceritinib (16.6 mo-NR) vs. chemotherapy (NE)	2017	[33]
ASCEND-5	III	Ceritinib vs. Single-agent chemotherapy	Progression following crizotinib and platinum-based doublet chemotherapy Patients with brain metastases	All BM: Ceritinib (n = 47) vs. chemotherapy (n = 48)	CNS ORR ceritinib (35%) vs. chemotherapy (5%) DOR ceritinib 6.9 mo (95% CI 2·7–8·3 vs. chemotherapy (not evaluable)	2017	[34]
NCT01970865	II	Lorlatinib	Treatment-naïve (cohort 1), progression on crizotinib (cohort 2), progression on crizotinib and chemotherapy (cohort 3), progression on non-crizotinib ALK inhibitor +/- chemotherapy, progression on 2 or 3 non-crizotinib ALK inhibitors +/- chemotherapy (cohort 4) Patients with asymptomatic BM (prior treatment allowed)	All BM: 141 Measurable BM: cohort 1 (n = 3), cohort 2 (n = 23), cohort 3 (n = 9), cohort 4 (n = 49)	CNS ORR cohort 1 (66.7%), cohort 2 (87%), cohort 3 (55.6%), cohort 4 (53.1%) Median DOR (months), cohort 1 [NR (NR–NR)], cohort 2 [NR (8·4–NR)], cohort 3 [NR (4·1–NR)],	2018, 2021	[35,36]
CROWN	III	Lorlatinib vs. Crizotinib	Treatment-naïve Patients with asymptomatic BM (prior treatment allowed)	All BM: Lorlatinib (n = 38) vs. Crizotinib (n = 40) Measurable BM: Lorlatinib (n = 17) vs. Crizotinib (n = 13)	All BM: CNS ORR, Lorlatinib (66%) vs. Crizotinib (20%); OR 8.41 (95% CI: 2.59–27.23) Measurable BM: CNS ORR, Lorlatinib (82%) vs. Crizotinib (23%); OR 16.83 (95% CI: 1.95–163.23) 12-month iPFS: Lorlatinib (95%) vs. Crizotinib (60%); HR 0.07 (95% CI: 0.03–0.17)	2020	[37]
***ROS1* Rearrangement**							
ALKA-372-001 STARTRK-1 STARTRK-2	I or II	Entrectinib	Treatment- naïve and previously treated Patients with asymptomatic or stable BM with pretreatment	All BM (n = 46) Measurable BM (n = 24)	All BM: CNS ORR, 52.2%; Median DOR: 12.9 mo; iPFS: 8.3 mo Measurable BM: CNS ORR, 79.2%; Median DOR: 12.9 mo; iPFS: 12 mo	2021	[38]
NCT01970865	II	Lorlatinib	Treatment- naïve and previously treated Patients with asymptomatic BM (prior treatment allowed)	Treatment-naïve (n = 11) Previously treated (n = 24)	Treatment-naïve: CNS ORR, 64%; Median DOR: NR (95% CI: 5.7 to NR) Previously treated: CNS ORR, 50%; Median DOR: NR (95% CI: 11.0 to NR)	2019	[39]
TRIDENT-1	II	Repotretinib	Treatment- naïve and previously treated Patients with asymptomatic BM (prior treatment allowed) and/or asymptomatic leptomeningeal disease included	Treatment- naïve (n = 3) Previously treated (n = 4)	Treatment- naïve: CNS ORR, 100% (3/3) Previously treated: CNS ORR, 50% (2/4)	2019	[40]
***KRAS* G12C**							
CodeBreak 100	II	Sotorasib	Previously treated Patients with active BMs were excluded	N = 26	No CNS results reported	2021	[41]
KRYSTAL-1	I	Adagrasib	Previously treated Patients with active and/or stable previously treated BMs included	N = 42	CNS ORR 33%	2022	[42]
***BRAF* V600E Mutation**							
NCT01336634	II	Dabrafenib and trametinib	Treatment -naïve and previously treated patients Patients with asymptomatic BM (if untreated, BM must be <1 cm, and if treated, must be stable for at least 3 weeks prior to enrollment)	All BM: (n = 3); Treatment naïve (n = 2) vs. Previously treated (n = 1)	Treatment- naïve group: (n = 2), best response of non-CR or non-PD reported Previously treated: (n = 1), N/A	2016, 2017	[43,44]
***NTRK*1/2/3 Gene Fusion**							
ALKA-372-001 STARTRK-1 STARTRK-2	I or II	Entrectinib	TRK inhibitor-naïve patients Patients with asymptomatic BMs (prior treatment allowed)	All BM: (n = 16) Measurable BM: (n = 8)	All BM: CNS ORR, 50%; Median iPFS: 8.9 mo Measurable BM: CNS ORR, 62.5%; Median iPFS 10.1 mo	2020	[45]
NCT02576431, NCT02637687	I or II	Larotrectinib	Non-primary CNS malignancy with BM or primary CNS malignancy Patients with asymptomatic BM	All BM: (n = 5) BM in lung cancer: (n = 3)	All BM: CNS ORR, 60%	2019	[46]
***MET* Exon 14 Skipping**							
GEOMETRY	II	Capmatinib	Treatment-naïve and previously treated patients Patients with non-enlarging BM (steroids therapy allowed, but no dose escalation in 2 weeks before enrollment)	All BM: (n = 13)	CNS ORR: 54%	2020	[47]
VISION	II	Tepotinib	Treatment-naïve and previously treated patients Patients with asymptomatic BM	All BM: (n = 15) Measurable BM: (n = 7)	CNS ORR: 71% (5/7) CNS DOR: 87% (13/15)	2022	[48]
***RET* Rearrangement**							
ARROW	I or II	Pralsetinib	Treatment-naïve and previously treated patients Patients with stable, non-enlarging BM and absence of neurologic symptoms	Measurable BM (n = 9)	CNS ORR: 56%, all complete responders	2021	[49]
LIBRETTO-001	I or II	Selpercatinib	Treatment-naïve and previously treated patients Patients with stable neurologic diseases at baseline (steroids allowed, 14 days before enrollment; no neurosurgery or radiation for 28 days; SRS allowed, 14 days before enrollment)	Measurable BM (n = 22)	Overall CNS ORR 81.8%; Prior RT (85.7%) vs. RT-naïve (75%) CNS Median DOR: 9.4 mo	2021	[50]
**ERBB2 (HER2) Mutation Positive**							
DESTINY-Lung01	II	Trastuzumab Deruxtecan	Patients with asymptomatic brain metastases Progression following standard of care	BM (n = 33)	PFS was 7.1 mo (95% CI, 5.5 to 9.8) and OS 13.8 mo (95% CI, 9.8 to 20.9) No CNS-specific outcomes	2022	[51]

Abbreviations: non-small cell lung cancer (NSCLC); progression of disease (POD); tyrosine kinase inhibitor (TKI); brain metastases (BM); central nervous system (CNS); objective response rate (ORR); odds ratio (OR); duration of response (DOR); intracranial progression-free survival (iPFS); progression-free survival (PFS); hazard ratio (HR); confidence interval (CI); standard of care (SOC); overall survival (OS); complete response (CR); not reached (NR); radiotherapy (RT).

**Table 2:** Efficacy of Immune Checkpoint Inhibitor (ICI) Monotherapy in Non-small Cell Lung Cancer (NSCLC) with Central Nervous System (CNS) Metastases.

Trial	Phase	Systemic Therapy	Setting/CNS Inclusion Criteria	Sample Size (with CNS Disease)	Key Results	Publication Year	Reference
NCT 02085070	II	Pembrolizumab	PD-1 or PD-L1 therapy-naïve Patients with >1 asymptomatic BM (5–20 mm) which was not previously treated or with unequivocal PD following local therapy	PD-L1 ≥ 1% (n = 37) PD-L1 < 1% (n = 5)	PD-L1 ≥ 1%: CNS ORR, 29.7% iPFS: 2.3 mo Median CNS DOR: 5.7 mo PFS: 1.9 mo OS: 9.9 mo PD-L1 < 1%: CNS ORR (0%) PFS: NR OS: NR	2020	[72]
OAK	III	Atezolizumab vs. Docetaxel	PDL-1 unselected, previously treated advanced or metastatic NSCLC Patients with asymptomatic treated BM	All BM: Atezolizumab (n = 61) vs. Docetaxel (n = 62)	Time to radiographic identification of new symptomatic BM: NR (Atezolizumab) vs. 9.5 mo (HR 0.38, 95% CI: 0.16–0.91) OS: 16.0 mo vs. 11.9 mo (HR = 0.74, *p* = 0.1633), atezolizumab vs. chemotherapy, respectively	2019	[77]
EMPOWER-Lung 1	III	Cemiplimab vs. Platinum doublet chemotherapy	Stage IIIB/C, IV squamous or nonsquamous NSCLC with PD-L1 > 50% Patients with asymptomatic BM (prior treatment allowed)	Cemiplimab (n = 34) vs. Chemotherapy (n = 34)	CNS outcomes not reported PFS: HR = 0.45 (favoring cemiplimab) OS: HR = 0.17 (favoring cemiplimab)	2021	[78]
FIR	II	Atezolizumab	Previously treated Patients with asymptomatic or treated brain metastases	Atezolizumab (n = 13)	CNS outcomes not reported sORR: 23% PFS: 4.3 mo OS: 6.8 mo	2018	[79]
Post hoc analysis of data from KEYNOTE-001, 010, 024, 042	Post hoc analysis	Pembrolizumab vs. Chemotherapy	Previously treated or untreated PD-L1 positive NSCLC Patients with asymptomatic BM	Pembrolizumab: (n = 199) Chemotherapy (n = 94)	PD-L1 ≥ 50% (IO vs. Chemo, respectively): sORR 33.9% vs. 4.6% PFS: 4.1 mo vs. 4.6 mo OS: 19.7 mo vs. 9.7 mo PD-L1 ≥ 1%: sORR: 26.1% vs. 18.1% PFS: 2.3 mo vs. 5.2 mo OS: 13.4 mo vs. 10.3 mo	2021	[80]

Abbreviations: brain metastases (BM); progressive disease (PD); central nervous system (CNS); objective response rate (ORR); intracranial progression-free survival (iPFS); duration of response (DOR); progression-free survival (PFS); overall survival (OS); not reached (NR); hazard ratio (HR); confidence interval (CI); systemic objective response rate (sORR).
